# Effect of Pouring Techniques and Funnel Structures on Crucible Metallurgy: Physical and Numerical Simulations

**DOI:** 10.3390/ma17194920

**Published:** 2024-10-08

**Authors:** Wenwen Feng, Wenkang Yao, Lin Yuan, Ye Yuan, Yiming Li, Pu Wang, Jiaquan Zhang

**Affiliations:** 1 School of Metallurgical and Ecological Engineering, University of Science and Technology Beijing, Beijing 100083, China; 2 Qingdao Yunlu Advanced Materials Technology Co., Ltd., Qingdao 266232, China

**Keywords:** planar flow casting, funnel structure, crucible, fluid flow, inclusion

## Abstract

In the planar flow casting process of amorphous strips, the flow behavior of molten metal and the inclusion content in the crucible are crucial to the morphology and magnetic properties of the material. This study conducts a comparative analysis of the effects of non-immersed and immersed funnels, as well as various funnel structures, on the fluid flow and inclusion removal efficiency in the crucible by integrating numerical and physical models. The findings reveal that for the same pouring flow rate, the diameter of the liquid column in non-immersed pouring conditions is smaller than that of the funnel outlet, leading to a faster injection flow velocity. As a result, the melt in the crucible is subjected to severe impacts, accompanied by an increased possibility of slag entrapment. Conversely, immersed pouring substantially reduces the velocity of the molten metal at the funnel outlet, thereby extending the residence time in the crucible and diminishing the volume of the dead zone. Additionally, the molten metal backflows due to the negative pressure formed in the inner chamber of the funnel. The design of a trumpet-shaped funnel increases the effective volume while reducing the height of the backflow fluid, consequently reducing the velocity of the molten metal at the funnel outlet and prolonging the residence time. Compared to the conventional pouring process with the non-immersed funnel, the outlet velocity is reduced from 1.1 m/s to 0.12 m/s by adopting the immersed funnel with an inverted trapezoidal trumpet structure. This reduction results in a stable flow state, a 9.69% reduction in the dead zone volume fraction, and a 22.96% increase in average inclusion removal efficiency. These improvements demonstrate that a crucible funnel with a well-designed structure and the implementation of an immersion process can significantly improve the metallurgical effects in the planar flow casting process.

## 1. Introduction

**Figure 1:**
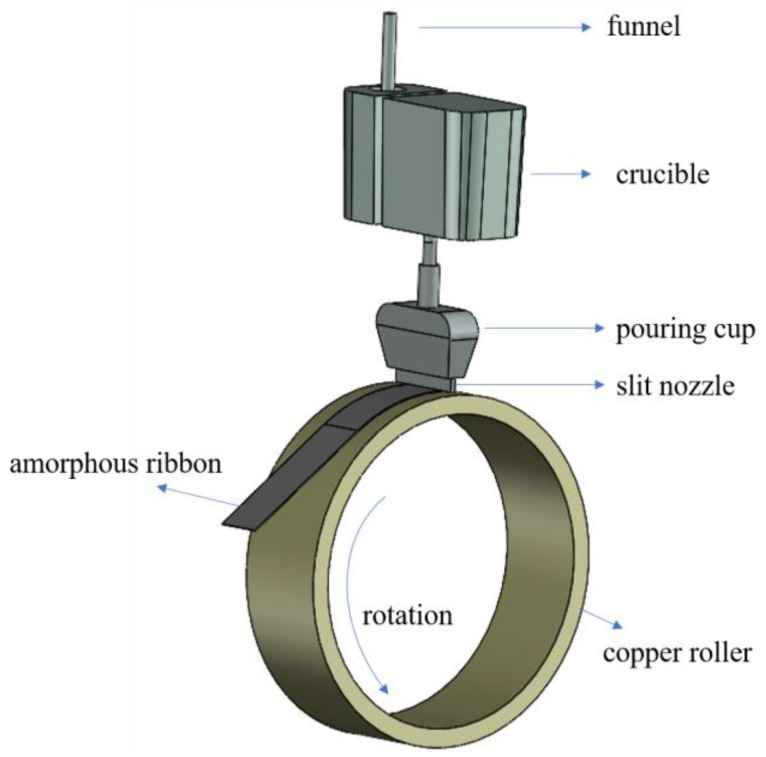
Diagram of the planar flow casting process (PFC).

As depicted in Figure 1, the planar flow casting process is an advanced rapid solidification process to prepare amorphous strips with cooling rates as high as 10^6^ K/s. Initially, the raw materials are heated to the melting point in the induction melting furnace and then refined by means of bottom-blown argon. The molten melt then enters the crucible through a receiving funnel, and is ejected onto the surface of a rotating copper roller at high velocity. The copper roller, in conjunction with a copper wheel, efficiently dissipates heat, facilitating the rapid solidification of the melt into amorphous thin strips. Following solidification, strips are carefully detached and conveyed through guide rolls to a winding device where they undergo coiling and preliminary inspection [1,2,3,4]. The crucible plays a critical role in the planar flow casting process by providing stable pouring conditions, which facilitates continuous casting, removes large inclusions, and alleviates secondary oxidation of the molten metal. This function is similar to the tundish used in steel production. Similarly, the purpose of the steel-receiving funnel is the same as that of the ladle shroud [5,6,7,8,9]. Notably, unlike in traditional continuous casting processes, non-immersed funnels are widely used in the planar flow casting of amorphous strips. While the attention to the design and optimization of the funnel and crucible specific to this process is still limited, the operational principles can draw valuable insights from existing research on the ladle shroud and tundish in steel processing.

To improve the quality of molten steel and minimize the contact of the molten steel with air, protective pouring techniques are employed during the continuous casting process [10,11]. The ladle shroud, serving as a critical conduit connecting the steel ladle and the tundish, is used for protected pouring between the steel ladle and the tundish. Utilizing a ladle shroud also helps reduce surface fluctuations in the tundish, minimizes slag entrapment, and improves the quality of the molten steel [12]. Lu [13] supposed that when the ladle shroud was not submerged below the surface of the molten steel, direct impacts on the steel surface could be avoided. However, the intense turbulence generated can cause fluctuations and splashing at the beginning of pouring, severely impacting the effectiveness of protective pouring. Conversely, a submerged ladle shroud, with its end immersed in the molten steel, significantly reduces steel splashing and oxidation, improving the stability of the molten steel and thus enhancing the internal quality of the product. Cui et al. [14] inserted the ladle shroud vertically about 100 mm into the molten steel surface to avoid the secondary oxidation caused by exposure and splashing of the molten steel due to the impact of the injection flow on the surface of the steel in the tundish. Moreover, the overall oxygen content and the heat loss of the molten steel could be controlled.

Different ladle shroud structures change the fluid flow in the impact zone of the tundish and throughout the entire tundish. Wen et al. [15] studied the stability of fluid flow patterns and the impact on the flow characteristics in the tundish using trumpet-shaped and straight-through ladle shrouds. Their findings indicated that the use of trumpet-shaped ladle shrouds resulted in a more stable flow field, which was beneficial for extending the response time and mean residence time of the fluid within the tundish, and for reducing the proportion of dead zones inside the tundish. Zhang et al. [16] compared straight-through and trumpet-shaped ladle shrouds and discovered that the trumpet-shaped shroud with larger outlet diameter and lower outlet velocity could significantly alleviate the erosion of refractory materials in the impact zone. Similarly, Yang et al. [17] observed that the greater inner diameter of the trumpet-shaped shroud effectively reduced the velocity of molten steel, thereby diminishing the turbulent energy in the impact zone. Zhang et al. [18,19,20] simulated the metallurgical effects of straight-through and trumpet-shaped ladle shrouds. The results showed that the reduced outlet velocity of the trumpet-shaped shroud diminished the impact on the molten melt in the tundish.

It is evident that the structural design and operation of the funnel in the planar flow casting process directly determine the characteristics of the outflow jet, namely the inflow characteristics of the crucible. These inflow characteristics are closely related to the turbulent state of the molten steel in the pouring area of the crucible, whether the molten metal is exposed, the occurrence of slag entrapment, and the intensity of erosion on the refractory materials. Additionally, the inflow characteristics of the funnel influence the overall flow and mixing state of the molten metal in the crucible, thereby affecting the effectiveness of inclusion removal. Inclusions, as non-metallic impurities, become stress concentration points within the material, reducing the strength and toughness of the amorphous strip material. When manufacturing transformer core materials, the presence of inclusions can disrupt the uniform distribution of magnetic flux, increase local magnetic resistance, and lead to a decrease in overall magnetic performance. Therefore, this paper employs numerical and physical models to study the flow behaviors of molten metal and inclusion removal efficacy in the crucible under non-immersed and immersed pouring processes and different funnel shapes. This research provides a theoretical basis for planar flow casting.

## 2. Model Description

**Figure 2:**
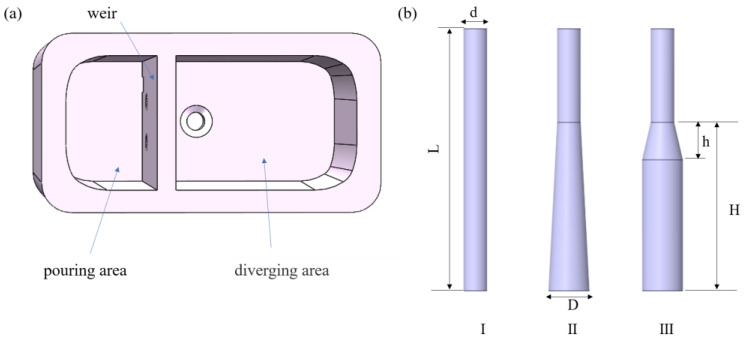
Schematic diagram of (**a**) the three-dimensional structure of the crucible and (**b**) the funnel structure, in which d is funnel inlet diameter, D is funnel outlet diameter, L is funnel length, h is horn section height, and H is expansion segment height.

Focusing on the crucible of the planar flow casting process, this study compares and analyzes the behavior of molten metal flow and inclusion removal under non-immersed and immersed pouring conditions, as well as under different funnel structures. To facilitate the comparison with the traditional straight-through funnel, two types of trumpet-shaped ladle shroud funnels were designed; the design followed the principle of H/D ≥ 4. This design criterion helps to effectively prevent backflow and achieve a more uniform distribution of outlet velocities [21]. Figure 2a,b present the three-dimensional structure of the crucible and the three different funnel structures. The relevant process parameters are listed in Table 1.

### 2.1. Water Model Experiment

Under isothermal flow conditions, the flow in the water model is similar to the flow of molten metal in the crucible when the Reynolds number (*Re*) and Froude number (*Fr*) remain constant [22,23]. Based on the principle of similarity, a water model with the similarity ratio of 1:1 was constructed.

(1) Frm=Frp=um 2gLm=up 2gLp

(2) $$Re_{m}=Re_{p}=\frac{\rho_{m}u_{m}L_{m}}{\mu_{m}}=\frac{\rho_{p}u_{p}L_{p}}{\mu_{m}}$$

The kinematic viscosities of water and the alloy liquid are comparable, approximately 10^−6^ (m^2^·s^−1^). Equations (1) and (2) can be derived.

(3) $$\frac{u_{m}}{u_{p}}={\left(\frac{L_{m}}{L_{p}}\right)}^{0.5}=\lambda^{0.5}$$

where *m* and *p* are model and prototype, u is the velocity of fluid (m·s^−1^), L is the characteristic length (mm), g is the acceleration of gravity (m·s^−2^), and λ is the similarity ratio.

Based on Equations (1)–(3), this leads to

(4) $$Q_{m}=\lambda^{\frac{5}{2}}Q_{p}$$

(5) $$t_{m}=\lambda^{\frac{1}{2}}t_{p}$$

where Q is flow rate (m^3^·h^−1^) and t is time (s).

**Figure 3:**
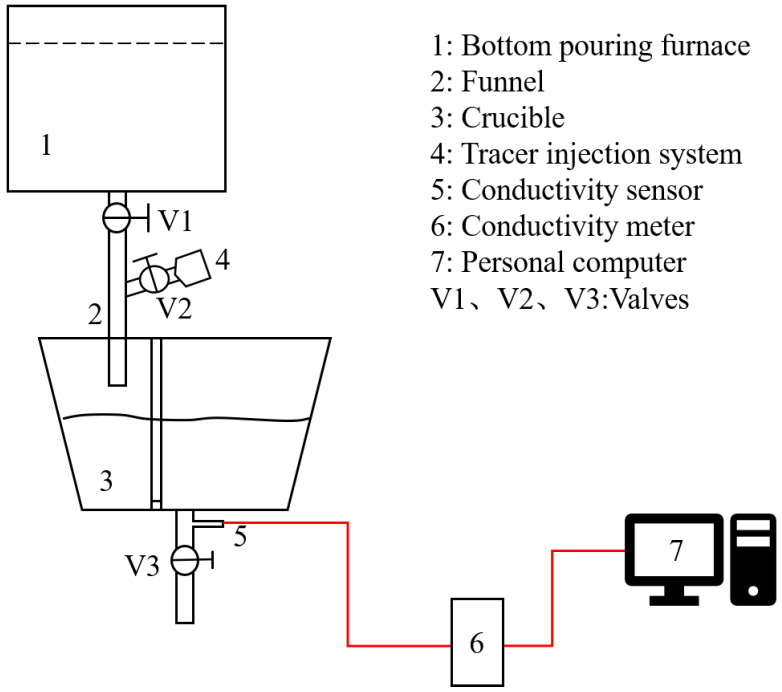
Schematic diagram of the water model experimental apparatus.

The experimental setup for the water model is depicted in Figure 3. Flow field tracing techniques and the exciting–responding method are utilized to document the diffusion of tracers within the crucible and to map out the residence time curves [24,25]. The method is as follows: In a steady-state flow, a stimulus signal (a tracer with physical properties similar to those of the original fluid) is injected into the inflow of the crucible at the instant of time *t* = 0. The tracer concentration *C*(t) is then continuously measured at the exit of the crucible. From the response curve, the distribution of the fluid’s residence time within the crucible is obtained.

The process for the water model experiment can be summarized as follows: (1) Eliminate the effect of volume changes on tracer results by adjusting the flow rates of V1 and V3 to maintain a constant crucible level. (2) When the liquid level is stabilized, inject 20 mL of a low-concentration potassium chloride solution into the crucible using a pulse from the tracer injection system, and the addition of potassium chloride solution can change the conductivity of the water in the crucible. (3) Monitor the changes in the electrical conductivity of the water at the crucible’s outlet using a conductivity meter and collect the data using a computer. To minimize experimental errors, each set of experiments is repeated three times. The relationship between dimensionless concentration and dimensionless time is obtained by conversion, which is an important tool used to evaluate the flow field inside the crucible. (4) Replace the solution with black ink and pulse it into the crucible, while simultaneously starting the camera to record flow process of the tracer. It enables visualization of the flow behavior within the crucible. (5) An amount of 2 g of polyethylene particles with a particle diameter of 5 μm is weighed using a microbalance, dissolved in 50 mL of anhydrous ethanol, and injected into a crucible via the tracer injection system. After waiting for 20 s, 500 mL of the solution is taken, filtered and dried, and weighed, and each set of experiments is repeated twice to take the average value to qualitatively compare the effect of inclusion removal under different optimization schemes. In addition, the mixed oil is utilized to simulate the slag.

### 2.2. Mathematical Model

#### 2.2.1. Assumptions

In the numerical simulation process, the assumptions of the model are outlined as follows:

1. The molten metal within the crucible is considered a steady-state, viscous, incompressible Newtonian fluid, with constant temperature and viscosity.
2. The flow of molten metal and air within the crucible is considered as three-dimensional, two-phase flow, without accounting for the influence of the slag layer.
3. The interface between the molten metal and the crucible walls is set as a no-slip boundary, meaning the velocity at the inner walls of the crucible is zero.

#### 2.2.2. Control Equations

(A) Fluid Flow Model

Fluid flow is described by the continuity equation and the momentum equation of the Euler–Lagrange method.

Continuity Equation:

(6) $$\frac{\partial \left(\rho u_{i}\right)}{\partial x_{i}}=0$$

Momentum Equation:

(7) $$\frac{\partial \left(\rho u_{i}\right)}{\partial t}+\rho \frac{\partial \left(u_{i}u_{j}\right)}{\partial x_{j}}=-\frac{\partial p}{\partial x_{i}}+\frac{\partial }{\partial x_{j}}\left[\mu_{\mathrm{eff}}\left(\frac{\partial u_{i}}{\partial x_{j}}+\frac{\partial u_{j}}{\partial x_{i}}\right)\right]+\rho g$$

where ρ is the fluid density (kg·m^−3^), μ is the liquid viscosity (kg·m^−1^·s^−1^), p is static pressure (N·m^−2^), and $\mu_{eff}$ is the effective viscosity coefficient (kg·m^−1^·s^−1^).

(B) Volume of Fluid (VOF) Model

In the VOF model, the sum of the volume fractions of each phase in every computational cell equals 1. The interface is tracked by solving the continuity equation for the phase volume fractions.

(8) $$\frac{\partial \alpha }{\partial t}+\vec{u}\cdot \nabla \alpha =0$$

The density and viscosity of the mixed fluid in each computational cell are calculated using Equations (9) and (10).

(9) $$\rho =\alpha \rho_{l}+(1-\alpha )\rho_{g}$$

(10) $$\mu =\alpha \mu_{l}+(1-\alpha )\mu_{g}$$

where $\rho_{l}$ and $\rho_{g}$ are the liquid and air density (kg·m^−3^), $\mu_{l}$ and $\mu_{g}$ are the liquid and air viscosity (kg·m^−1^·s^−1^), and α is the volume fraction of liquid.

(C) Turbulence Model

The standard k–ε turbulence model was employed to simulate the flow field of molten steel. The equations for turbulent kinetic energy and turbulence dissipation rate are presented as Equations (11)–(13).

Turbulence Model Equation:

(11) $$\frac{\partial \left(\rho ku_{i}\right)}{\partial x_{i}}=\frac{\partial }{\partial x_{i}}\left(\frac{\mu_{\mathrm{eff}}}{\sigma_{k}}\times \frac{\partial k}{\partial x_{j}}\right)+G_{k}-\rho \varepsilon$$

(12) $$\frac{\partial \left(\rho \varepsilon u_{i}\right)}{\partial x_{i}}=\frac{\partial }{\partial x_{i}}\left(\frac{\mu_{\mathrm{eff}}}{\sigma_{e}}\times \frac{\partial \varepsilon }{\partial x_{i}}\right)+C_{1\varepsilon }\frac{\varepsilon }{k}G_{k}-C_{2\varepsilon }\rho \frac{\varepsilon^{2}}{k}$$

(13) $$G_{k}=\mu_{t}\frac{\partial u_{j}}{\partial x_{i}}\left(\frac{\partial u_{i}}{\partial u_{j}}+\frac{\partial u_{j}}{\partial u_{i}}\right)$$

where $G_{k}$ is the turbulent energy term; k is the turbulent kinetic energy (m^2^·s^2^); ε is the turbulent kinetic energy dissipation rate (m^2^·s^3^); and $C_{1\varepsilon }$, $C_{2\varepsilon }$, $\sigma_{k}$, and $\sigma_{\varepsilon }$ from Launder [26] are 1.44, 1.92, 1.0, and 1.3.

(D) Species Transport Model

The RTD curve of the crucible outlet was monitored by adding tracer to the molten steel, and the flow behavior of the molten steel was described mathematically.

Component Transport Equation:

(14) $$\frac{\partial \left(C\rho \right)}{\partial t}+\frac{\partial \left(C\rho u_{i}\right)}{\partial x_{i}}=\frac{\partial }{\partial x_{i}}\left(\rho D_{\mathrm{eff}}\frac{\partial C}{\partial x_{i}}\right)$$

where C is the component volume concentration and $D_{eff}$ is the component diffusion coefficient (m^−2^·s^−1^).

After the RTD curve was obtained, the analysis and key parameters such as the mean residence time and the volume fraction of the dead zone were implemented [27,28].

(15) $${\bar{t}}_{c}=\frac{\int_{0}^{\infty }Ctdt}{\int_{0}^{\infty }Cdt}$$

(16) $$V_{p}=\frac{t_{\min}+t_{\max}}{2t_{i}}$$

(17) $$V_{d}=1-\frac{{\bar{t}}_{c}}{t_{i}}$$

(18) $$V_{m}=1-V_{p}-V_{d}$$

where ${\bar{t}}_{c}$ is the mean residence time (s); $V_{P}$, $V_{d}$, and $V_{m}$ are the volume fractions of the plug, dead, and mixed zones; $t_{min}$ and $t_{peak}$ are response time and peak concentration time (s), respectively; and $t_{i}$ is the theoretical mean residence time (s).

(E) Inclusion motion model

After calculating the multiphase model, the air domain is removed, leaving only the molten metal phase, and the upper surface is set as a wall. The Discrete Phase Model (DPM) is used to simulate the trajectories of non-metallic inclusion particles within the crucible. Once the system reaches a steady state, inclusions are uniformly added across the cross-section of the funnel entrance. These inclusion particles are modeled as spherical and interactions between particles are neglected, as well as their influence on the fluid.

Particle Transport Equation:

(19) $$\frac{du_{P}}{dt}=F_{D}(\vec{u}-\vec{u_{P}})+\frac{g(\rho_{p}-\rho )}{\rho_{p}}+\vec{F}$$

where $F_{D}$ is the drag force per unit of mass of the particles, $u_{p}$ represents the velocity of particle (m·s^−1^), and $\rho_{p}$ is the density of the particle (kg·m^−3^). The parameter $F_{D}$ is calculated as a function of the relative Reynolds number given by the difference in velocities between the continuous phase and the particle [29], and $\vec{F}$ is an additional acceleration term, including Saffman lift force, virtual mass force, and pressure gradient force.

#### 2.2.3. Boundary Conditions and Solution

The crucible inlet and outlet are both set as velocity inlet boundary conditions. The free surface is established by setting the shear stress at the top surface of the crucible to zero in all directions. Standard wall functions are applied to the crucible walls, which are subject to a no-slip boundary condition. Inclusions of five distinct sizes are injected at the same velocity as the molten metal from the inlet. Inclusion collisions and growth are not taken into account.

**Figure 4:**
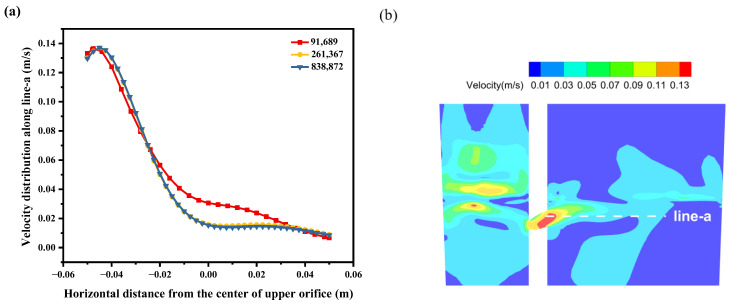
Schematic diagram of (**a**) velocity distribution of molten metal in the crucible at different mesh counts and (**b**) velocity distribution contour of longitudinal section (X = 36 mm).

The Fluent meshing 2022 R1 software is used to generate a Poly-Hexcore mesh in the model, and the Body Of Influence (BOI) method is used for localized refinement in areas with significant velocity gradients, such as the inlet and casting level. This refinement aims to enhance computational accuracy. The minimum mesh size in these refined areas is 2.5 mm, while the rest of the model maintains a minimum mesh size of 5 mm. Figure 4a compares the velocity distribution along monitoring line a in the crucible at different mesh numbers, and Figure 4b shows the position of monitoring line a. As shown in the figure, the velocity distribution on the monitoring line becomes more consistent as the number of meshes increases; the absolute errors of the ensemble average values of the calculation results of line a between each grid are 10.8 and 0.54 pct, respectively. To reduce the computational load, the mesh with 261,367 grids was used in this study.

In the computational process, the flow field within the crucible is first solved under steady-state conditions using the Coupled algorithm. The pressure term is set using the PRESTO! scheme, which can effectively deal with numerical errors caused by mesh distortion and enhance numerical stability. The other equations are discretized using a first-order upwind implicit scheme for solution. The hybrid initialization method is employed, with patch operations applied to the molten metal within the funnel. The simulation is considered converged when the residuals drop below 10^−4^. Subsequently, transient analyses are conducted to determine the concentration changes of tracers at the outlet.

Furthermore, to assess the removal efficiency of inclusions, air terms are omitted, the crucible’s liquid surface is treated as a wall, and the flow field calculations are repeated. Following this, the DPM is activated with the upper surface set to trap conditions to evaluate the removal of inclusions within the crucible. The physical and process parameters used in the numerical simulations are listed in Table 2, and the computational case is detailed in Table 3.

## 3. Results

### 3.1. Model Validation

**Figure 5:**
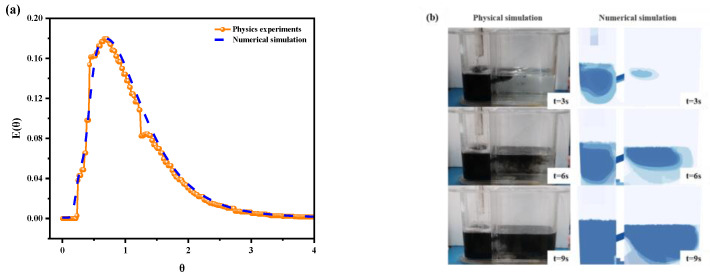
Comparison between physical and numerical simulations: (**a**) RTD curves, in which θ is dimensionless time and E(θ) is the probability density function. (**b**) Tracer flow patterns at different times.

To validate the reliability of the mathematical model, validations were conducted between the RTD (Residence Time Distribution) curves from both physical simulation experiments and numerical simulations, along with tracer flow patterns at different time intervals. Figure 5a indicates that the RTD curves from the physical experiments and numerical calculations show strong alignment and consistent trends. Figure 5b illustrates the tracer distributions at various times from the water model experiments and numerical simulations. At 3 s after the start of the ink-tracing experiment, the ink first flows out from the upper hole and reaches the right chamber. At 6 s, the ink passing through the upper hole diffuses towards the right wall at a certain speed along the free liquid surface, and ink begins to pass through the bottom hole. At the same time, influenced by gravity, the ink at the surface spreads towards the bottom. At 9 s, the ink reaches the outlet, covering most of the right chamber except near the weir. This comparison reveals that the tracer flow trajectories from the experimental and computational results are fundamentally consistent, thereby affirming the reliability and accuracy of the developed numerical model.

### 3.2. Analysis of Pouring Conditions

**Figure 6:**
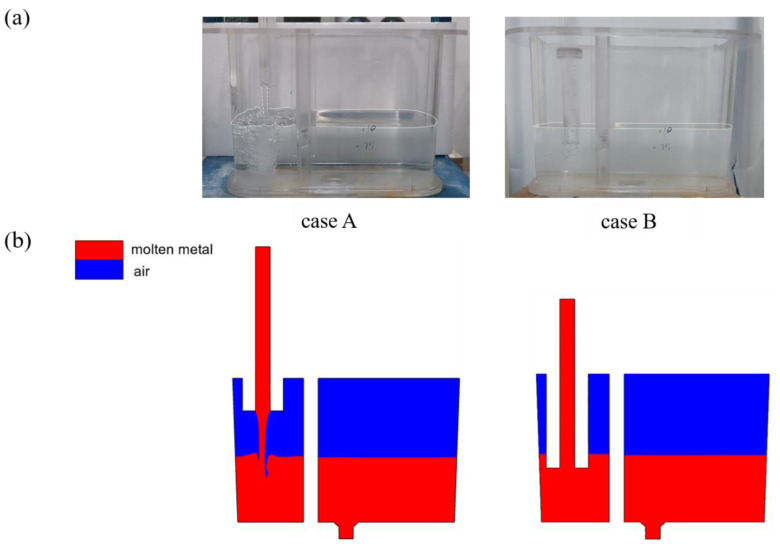
Phase distribution within the crucible during non-immersion and immersion casting: (**a**) water simulation (with white line indicating the water level); (**b**) numerical simulation.

**Figure 7:**
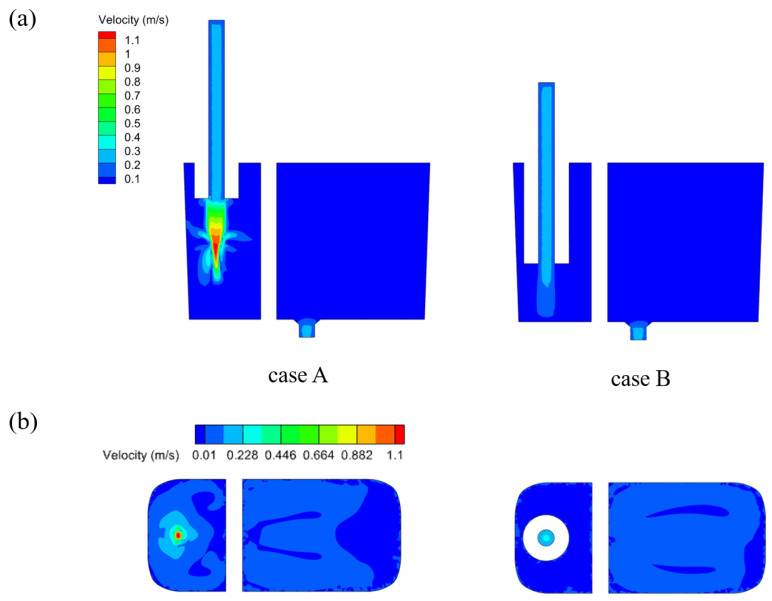
Velocity distribution within the crucible during non-immersion and immersion casting: (**a**) longitudinal cross-section at the funnel center; (**b**) target liquid level cross-section.

Figure 6 and Figure 7 show the phase and velocity distributions within the crucible under different pouring conditions. In case A, where the funnel is not submerged below the liquid surface, the molten metal exits as a slender stream with an average diameter of 10 mm (smaller than the funnel outlet diameter of 25 mm), impacting the liquid surface at a maximum velocity of 1.1 m/s. This high velocity results in a significant overall speed at the surface, causing air from the blue region to be entrained below the target liquid level. The exposed end of the funnel leads to the gas entering the left chamber of the crucible at high speed, causing severe surface fluctuations and generating numerous bubbles. In contrast, in case B where the funnel is submerged below the liquid surface, the fluid within the funnel provides significant buffering and deceleration, reducing the maximum fluid velocity to 0.3 m/s. The free liquid surface remains stable, with an overall maximum flow velocity of 0.06 m/s, leading to minimal bubble entrainment. This comparison illustrates that, at equivalent flow rates, non-immersion casting produces a slender, fast-moving flow. In contrast, immersion casting initially generates a rapid fluid flow, which creates negative pressure near the upper part of the funnel. This prompts the molten metal within the crucible to backflow and ascend within the funnel. Subsequently, the fluid velocity decreases [15].

**Figure 8:**
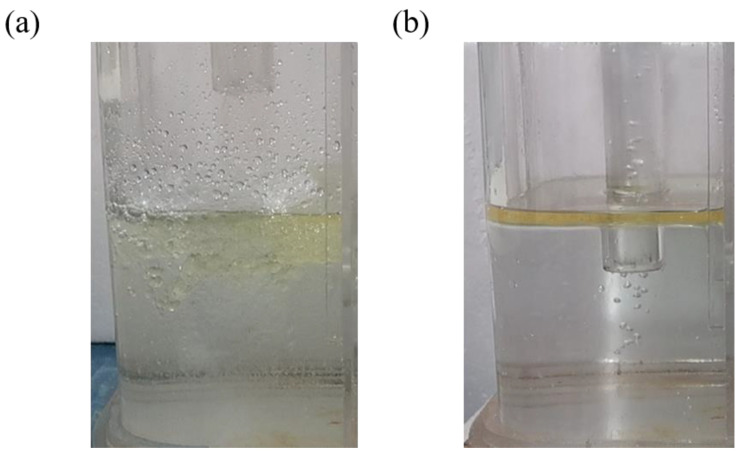
Fluctuation in steel–slag interface under different pouring conditions: (**a**) case A; (**b**) case B.

Figure 8 shows the fluctuations in the steel–slag interface in the left chamber of the crucible during water simulation experiments under different pouring conditions. In case A, the oil layer is damaged under strong impact, breaking into many small droplets that are driven below the liquid surface. If these droplets are not subsequently removed by flotation, they could form large inclusions, potentially affecting the quality of the strip. However, in case B, the oil layer remains stable, exhibiting only minor fluctuations without any mixing phenomena. This stability effectively isolates the molten metal from air, preventing oxidation and the formation of inclusions, thereby promoting the cleanliness of the molten metal [30,31].

**Figure 9:**
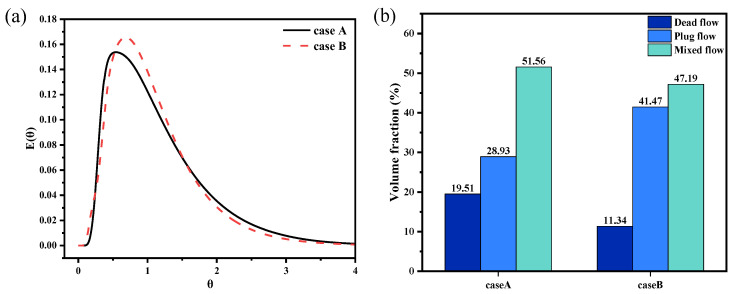
Flow characteristics of the crucible under different cases: (**a**) RTD curves, in which θ is the dimensionless time and E(θ) is the probability density function. (**b**) Mixing characteristics.

Figure 9 shows the RTD curves and the calculated flow characteristic parameters for various pouring cases. The data indicate that, under non-immersion casting conditions, the response time of the tracer is 8.4 s, with a peaking time of 28 s. However, after the funnel is immersed, the peaking time increases by 15 s, thereby extending the residence time within the tundish. Additionally, immersing the funnel appropriately below the liquid surface reduces the dead zone volume. Compared to case A, the dead volume and mixed flow volume in case B decrease by 8.17% and 4.37%, respectively, while the plug flow volume increases by 12.54%. This finding suggests that immersion casting not only enhances overall fluid dynamics within the crucible but also facilitates the collision, growth, and removal of inclusions through flotation.

### 3.3. Analysis of Funnel Shape Analysis

Comparative analyses of flow stream morphologies, velocity profiles, steel–slag interface fluctuations, RTD curves, and mixing characteristics under various pouring conditions indicate that submerging the crucible funnel into the fluid markedly reduces flow velocity, minimizes disturbances to the free liquid surface caused by the flow streams, and prolongs the fluid’s residence time within the crucible. Considering actual production conditions, the elevated air pressures generated by the straight-through crucible funnel immersion pouring technique can lead to the splashing of molten steel, potentially resulting in accidents. Consequently, two trumpet-shaped funnels were designed to evaluate the impact of funnel shape on fluid flow under immersion pouring conditions. Cases C and D both incorporate trumpet-shaped funnels; however, the type II funnel features a continuously expanding channel throughout its horn section, while type III expands to a larger diameter initially and maintains this diameter up to the outlet.

**Figure 10:**
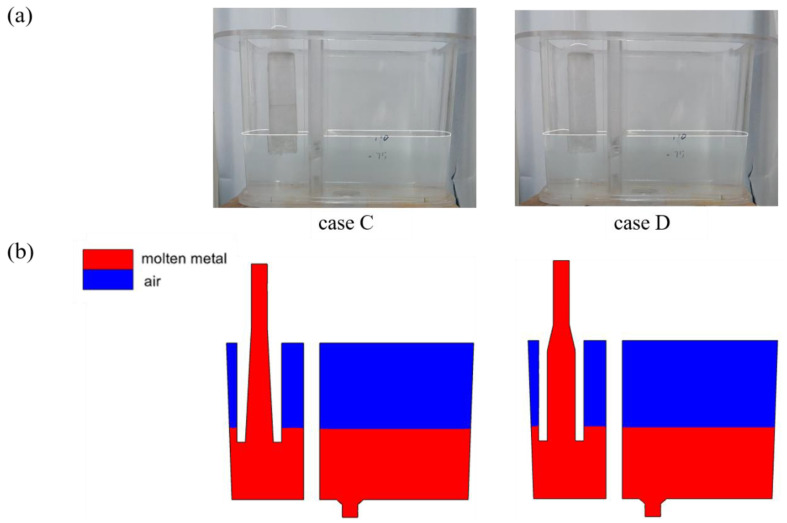
Phase distribution within the crucible under different trumpet-shaped funnels: (**a**) water simulation (with white lines indicating the water level); (**b**) numerical simulation.

Figure 10 shows the phase distribution within the crucible under different trumpet-shaped funnel configurations. As shown in the figure, when the funnel is immersed below the free liquid surface, negative pressure causes fluid to backflow into the funnel to a certain height. Since case D has a larger internal volume than case C, the resulting pressure head is lower. Compared to the straight-through funnel, no significant bubbles are observed beneath the trumpet-shaped nozzles, and the free liquid surfaces in the left chambers remain stable.

**Figure 11:**
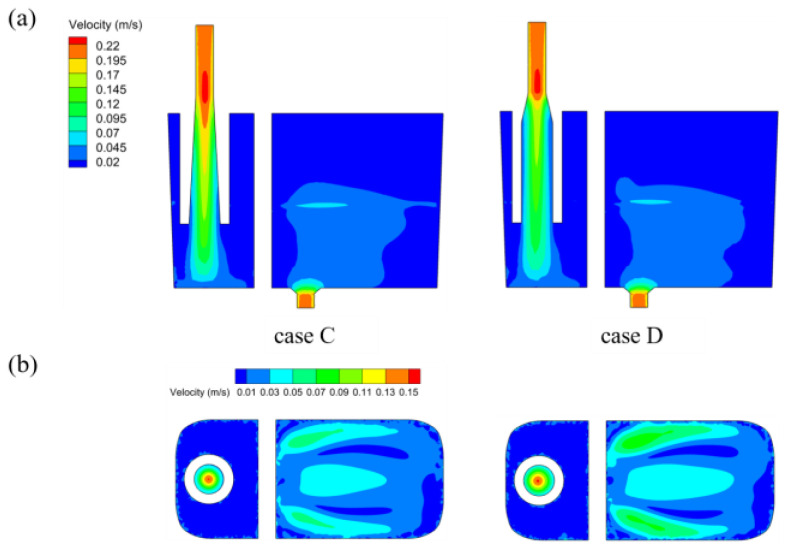
Longitudinal section and free surface velocity contours of the crucible under different trumpet-shaped funnels: (**a**) at the central cross-section of the funnel; (**b**) at the targeted liquid level section.

**Figure 12:**
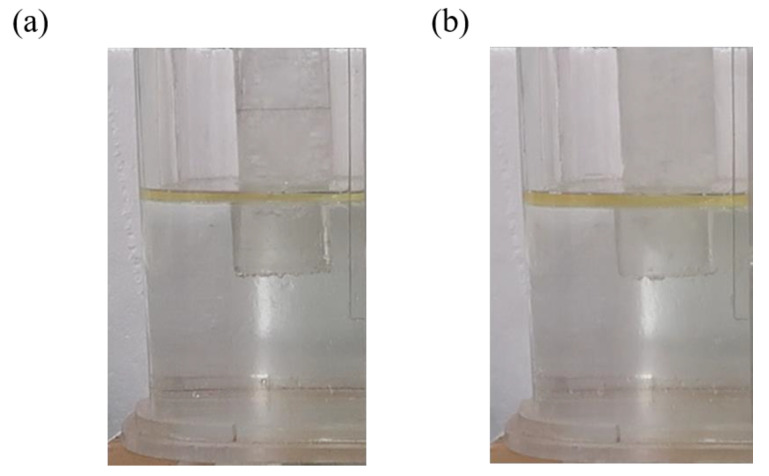
Fluctuation in steel–slag interface under different trumpet-shaped funnels: (**a**) case C; (**b**) case D.

Figure 11 shows the longitudinal cross-sections of the crucible and the velocity contours of the free liquid surface with different trumpet-shaped funnels, while Figure 12 shows the fluctuations in the steel–slag interface in the crucible’s left chamber. In contrast to the outlet velocity of 0.3 m/s observed with the straight funnel under immersion conditions, the flow velocities through the expansion sections of the trumpet-shaped funnels are significantly reduced, reaching approximately 0.14 m/s and 0.12 m/s, respectively. Furthermore, the maximum flow velocity in the right chamber remains below 0.1 m/s, ensuring an extremely stable oil layer structure devoid of any waves or mixing phenomena.

**Figure 13:**
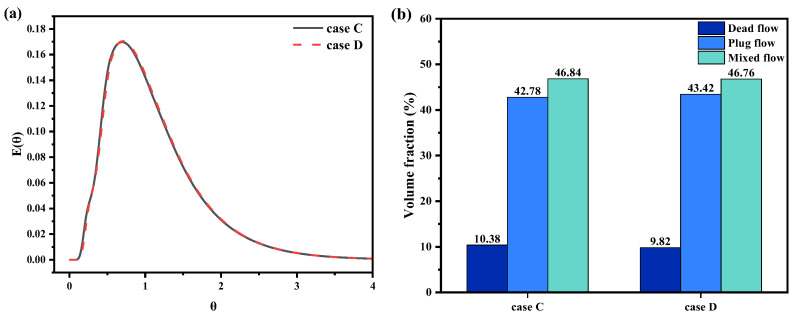
Flow characteristics of the crucible under different trumpet-type funnels: (**a**) RTD curves, in which θ is dimensionless time and E(θ) is the probability density function. (**b**) Mixing characteristics.

Figure 13 shows the RTD curves alongside the computed flow characteristic parameters for various trumpet-shaped funnel designs. The RTD curves for cases C and D exhibit substantial alignment, with their peaking times occurring nearly concurrently. Analyzing the mixing characteristics, it can be seen that, compared with the straight-through and type II funnel, the crucible dead flow volume fraction decreases to 9.82% with the type III funnel, which can further improve the cleanliness of the crucible’s molten metal.

### 3.4. Analysis of Inclusion Results

In the manufacturing process of amorphous strips, the quality is critically determined by the concentration and spatial distribution of inclusions. The crucible plays a pivotal role in metallurgical operations, being primarily responsible for the elimination of inclusions from the molten metal. The volume and granulometric distribution of these inclusions are key indicators of the melt’s metallurgical purity [32]. Therefore, the effects of immersion depth and funnel type on inclusion removal were analyzed using a combination of water modeling experiments and numerical simulations.

**Figure 14:**
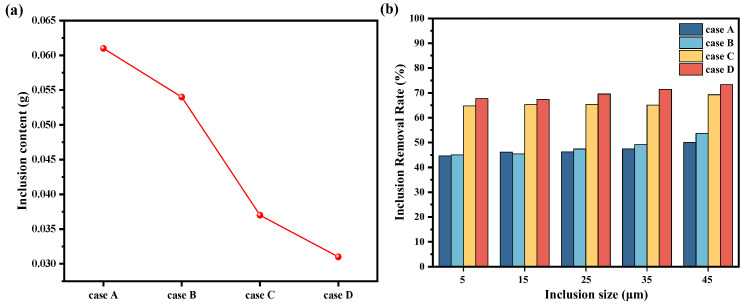
Inclusion removal rates under different cases: (**a**) physical simulation; (**b**) numerical simulation.

Figure 14 shows the removal rates of inclusions under different cases, as determined by both physical and numerical simulations. The figure shows that compared to non-immersion casting processes, immersion casting can reduce the escape rate of inclusions. Furthermore, based on the principles of immersion casting, the use of trumpet-shaped funnels can further decrease the escape rate of inclusions. Across the four cases, the overall removal rates increase with the diameter of the inclusion particles. This is due to the fact that larger particles are subjected to higher buoyancy forces, which facilitates the ascent to the steel–slag interface, where they are readily absorbed and removed. Consequently, removal rates increase as the size of the inclusions increases [33]. Among the cases, under immersion conditions, case D employing the type III funnel demonstrates the most effective inclusion removal. Physical simulations captured only 0.031 g of polyethylene particles, while numerical simulations indicated an average removal rate of 69.87% for inclusions across five particle sizes. Compared to case A, the amount of polyethylene particles collected by filtration decreased by 0.030 g, and the average removal rate of inclusions increased by 22.96%.

## 4. Conclusions

This study investigates the impact of different pouring conditions and funnel shapes on the metallurgical behavior of the crucible for the planar flow casting process, using a combination of numerical simulation and physical modeling. The main conclusions are as follows:

1. When using a straight-through funnel under non-immersion pouring conditions, the diameter of the liquid column remains smaller than the funnel’s outlet diameter. This condition leads to a pouring velocity of 1.1 m/s, which significantly impacts the molten melt in the crucible and increases the risk of slag entrainment. Furthermore, the molten metal within the crucible exhibits a relatively short mean residence time, with the dead zone ratio amounting to 19.51%.
2. When using a straight-through funnel, immersion pouring results in the liquid column diameter being equal to that of the funnel outlet, which reduces the molten metal flow velocity at the funnel exit to 0.3 m/s. This decrease in flow velocity extends the residence time of the molten metal within the crucible and reduces the dead volume fraction to 11.34%. However, the rapid fluid motion near the upper part of the funnel generates negative pressure, leading to the backflow of molten metal into the funnel, subsequently filling it. This phenomenon renders the approach unsuitable for practical production.
3. During immersion pouring using trumpet-shaped funnels, types II and III exhibit outlet velocities of 0.14 m/s and 0.12 m/s, respectively. The free liquid surface remains stable without any fluctuations or mixing phenomena, and the pressure head within the funnel cavity is reduced. Compared to case A, the use of the type III trumpet-shaped funnel decreases the dead volume fraction to just 9.69%. Physical simulations of polyethylene particle filtering showed a reduction of 0.030 g, and mathematical simulations determined an average inclusion removal rate improvement of 22.96%.

The pouring process and funnel structure significantly affect the flow of molten metal and the removal of inclusions during the planar flow casting process of amorphous strips. Specifically, the combination of immersion pouring with trumpet-shaped funnels markedly improves the flow behavior of the molten metal and enhances the efficiency of inclusion removal. This study ensures the castability of amorphous strips, which is beneficial for subsequent control over the quality, strength, toughness, and magnetic properties of the strip products.

## Figures and Tables

**Table 1:** Geometric structure and process parameters.

Parameters	Values
Liquid depth	110 mm
Capacity	0.043 T
Funnel flow rate	5.85 L·min^−1^
Funnel inlet diameter (d)	25 mm
Funnel outlet diameter (D)	44 mm
Length of funnel (L)	280 mm
Height of horn section (H)	180 mm
Height of expansion segment (h)	40 mm

**Table 2:** The parameters used in the mathematical model.

Parameters	Values
$\mathrm{Density}\;\mathrm{of}\;\mathrm{molten}\;\mathrm{metal}\;\left(\rho_{l}\right)$	7175 kg/m^3^
$\mathrm{Viscosity}\;\mathrm{of}\;\mathrm{molten}\;\mathrm{metal}\;\left(\mu_{l}\right)$	0.01 kg·m^−1^·s^−1^
$\mathrm{Density}\;\mathrm{of}\;\mathrm{air}\;\left(\rho_{g}\right)$	1.225 kg/m^3^
$\mathrm{Viscosity}\;\mathrm{of}\;\mathrm{air}\;\left(\mu_{g}\right)$	1.7894 × 10^−5^ kg·m^−1^·s^−1^
$\mathrm{Density}\;\mathrm{of}\;\mathrm{inclusion}\;\left(\rho_{p}\right)$	2700 kg/m^3^
$\mathrm{Diameter}\;\mathrm{of}\;\mathrm{inclusion}\;\left(d_{p}\right)$	5, 15, 25, 35, 45 μm
$\mathrm{Density}\;\mathrm{of}\;\mathrm{tracer}\;\left(\rho \right)$	7175 kg/m^3^
$\mathrm{Viscosity}\;\mathrm{of}\;\mathrm{tracer}\;\left(\mu \right)$	0.01 kg·m^−1^·s^−1^
$\mathrm{Specific}\;\mathrm{heat}\;\mathrm{capacity}\;\mathrm{of}\;\mathrm{tracer}\;\left(c_{p}\right)$	1350 J·kg^−1^·K^−1^
$\mathrm{Thermal}\;\mathrm{conductivity}\;\mathrm{of}\;\mathrm{tracer}\;\left(\lambda_{p}\right)$	42 W·m^−1^·K^−1^

**Table 3:** Details of computational cases.

Case	Immersion Depth (mm)	Funnel Type
A	−80	I
B	20	I
C	20	II
D	20	III

## Data Availability

The original contributions presented in the study are included in the article; further inquiries can be directed to the corresponding authors.
